# A Novel Highly Potent Therapeutic Antibody Neutralizes Multiple Human Chemokines and Mimics Viral Immune Modulation

**DOI:** 10.1371/journal.pone.0043332

**Published:** 2012-08-17

**Authors:** Michelle L. Scalley-Kim, Bruce W. Hess, Ryan L. Kelly, Anne-Rachel F. Krostag, Kurt H. Lustig, John S. Marken, Pamela J. Ovendale, Aaron R. Posey, Pamela J. Smolak, Janelle D. L. Taylor, C. L. Wood, David L. Bienvenue, Peter Probst, Ruth A. Salmon, Daniel S. Allison, Teresa M. Foy, Carol J. Raport

**Affiliations:** 1 Department of Protein Engineering, VLST Corporation, Seattle, Washington, United States of America; 2 Department of Immunology and Preclinical Pharmacology, VLST Corporation, Seattle, Washington, United States of America; 3 Department of Protein Sciences, VLST Corporation, Seattle, Washington, United States of America; French National Centre for Scientific Research, France

## Abstract

Chemokines play a key role in leukocyte recruitment during inflammation and are implicated in the pathogenesis of a number of autoimmune diseases. As such, inhibiting chemokine signaling has been of keen interest for the development of therapeutic agents. This endeavor, however, has been hampered due to complexities in the chemokine system. Many chemokines have been shown to signal through multiple receptors and, conversely, most chemokine receptors bind to more than one chemokine. One approach to overcoming this complexity is to develop a single therapeutic agent that binds and inactivates multiple chemokines, similar to an immune evasion strategy utilized by a number of viruses. Here, we describe the development and characterization of a novel therapeutic antibody that targets a subset of human CC chemokines, specifically CCL3, CCL4, and CCL5, involved in chronic inflammatory diseases. Using a sequential immunization approach, followed by humanization and phage display affinity maturation, a therapeutic antibody was developed that displays high binding affinity towards the three targeted chemokines. *In vitro,* this antibody potently inhibits chemotaxis and chemokine-mediated signaling through CCR1 and CCR5, primary chemokine receptors for the targeted chemokines. Furthermore, we have demonstrated *in vivo* efficacy of the antibody in a SCID-hu mouse model of skin leukocyte migration, thus confirming its potential as a novel therapeutic chemokine antagonist. We anticipate that this antibody will have broad therapeutic utility in the treatment of a number of autoimmune diseases due to its ability to simultaneously neutralize multiple chemokines implicated in disease pathogenesis.

## Introduction

Chemokines and their receptors play a central role in the immune system through mediating trafficking of leukocytes [1]. Chemokine signaling has been found to have homeostatic functions involved in tissue-specific recruitment of leukocytes as well as proinflammatory functions involved in induced recruitment of leukocytes initiated by inflammatory stimuli [2]. To date, ∼20 chemokine receptors and ∼50 chemokines have been identified. Regulation of this complex network arises from differential expression of chemokine receptors on leukocyte sub-populations and temporal expression of chemokines and their receptors during an inflammatory response. A central feature of chemokine biology is the redundancy present in the system as several chemokines are capable of binding a single receptor and *vice versa*. For example, CCL3 and CCL5 are both capable of inducing chemotaxis through binding of CCR1 and CCR5 receptors, while both CCR1 and CCR5 have additional non-redundant chemokine ligands.

A hallmark of chronic inflammatory diseases, including multiple sclerosis (MS) and rheumatoid arthritis (RA), is excessive infiltration of immune cells into specific tissue sites, ultimately resulting in tissue damage [3], [4]. While several strategies aimed at ameliorating disease focus on reducing the activity of the infiltrating immune cells through inhibition of activation (i.e., targeting co-stimulation), or neutralization of proinflammatory activity (i.e.,TNF α inhibitors), it also follows that preventing migration of leukocytes to the sites of inflammation may be an alternative strategy for treating inflammatory diseases. The therapeutic benefit of inhibiting leukocyte trafficking has been demonstrated by the efficacy of Tysabri, an antibody which inhibits leukocyte adhesion and subsequent recruitment into the central nervous system through binding α_4_-integrins, in the treatment of MS [5]. Similarly, inhibiting chemotaxis by targeting either the chemokine ligand or chemokine receptor may also prove to be efficacious, as evidenced by intensive efforts to identify chemokine ligands and receptors that may be involved in disease pathogenesis, as well as inhibitors of chemokine function.

While several chemokine receptors and ligands have been implicated in inflammatory disorders, mounting evidence has highlighted the relevance of CCR1 and CCR5 and their primary ligands (CCL3 and CCL5 for CCR1; CCL3, CCL4, and CCL5 for CCR5) in RA and MS. CCR1, a prominent receptor on monocytes, CCR5, a prominent receptor on T cells, including regulatory T cells, and tissue macrophages, and their primary ligands have been found to be abundantly expressed at the site of inflammation in both RA [3], [6]–[8] and MS [9]–[13]. Alternative CCR1 ligands, CCL15 and CCL23, are activated through proteolysis and are also found at high levels in synovial fluid [14]. Numerous studies in animal models demonstrate the disease relevance of CCR1 and CCR5-dependent cell migration, yet the complexity of the chemokine system has made it difficult to translate scientific discoveries into therapeutic treatments for inflammatory disorders [15]–[20]. This is evidenced by the fact that, to date, blockade of single chemokines or single chemokine receptors with small molecule or antibody inhibitors have demonstrated little or no activity in MS or RA clinical trials (see [21] for review). Redundancy in the chemokine/chemokine receptor network is often cited as a reason for the failure of chemokine antagonists in clinical studies [21].

While the relevance of redundancy within chemokine signaling is actively debated [22], it is interesting to note that both pox and herpes viruses have evolved soluble chemokine-binding proteins that are capable of binding multiple chemokines despite low sequence homology amongst the chemokines, suggesting the presence of evolutionary pressure to inhibit multiple chemokines [23], [24]. Specifically, Vaccinia and related pox viruses produce a soluble 35 kDa protein termed vCCI which binds and inhibits the majority of CC chemokines, including CCR1 and CCR5 ligands, presumably to evade host immune responses [25], [26]. The family of CC chemokines acts primarily on monocytes and T cells and is generally involved in chronic inflammation and in the host response to viral infection. Blocking a large set of CC chemokines may be necessary for complete inhibition of chronic inflammatory events, as demonstrated by the effectiveness of recombinant vCCI administration in reducing leukocyte infiltration in several mouse models of autoimmune disease, including collagen induced arthritis (CIA) [27] and experimental autoimmune encephalomyelitis (EAE) [28]. However, despite the efficacy of vCCI in animal models of disease, the likely immunogenicity of a viral protein and poor pharmacokinetic properties of vCCI present significant challenges to the use of vCCI itself as a therapeutic drug in humans.

Taking a cue from viral vCCI, we set out to develop therapeutic antibodies that also bind multiple CC chemokines, with the notion that a superior chemokine-targeting therapeutic for treatment of autoimmune disease would be one that inhibited multiple CC chemokines. Due to the inherent difficulties in mimicking the breadth of vCCI binding specificity, we targeted a subset of vCCI ligands that have been shown to be relevant in inflammatory disorders, the primary ligands for CCR1 and CCR5 (CCL3, CCL4, and CCL5). Here we describe the development and characterization of a therapeutic antibody that potently inhibits CCL3, CCL4, and CCL5. This antibody inhibits chemokine activities on receptors CCR1 and CCR5 both *in vitro* and *in vivo* and represents a novel class of chemokine inhibitor as a potential treatment for human autoimmune diseases.

## Materials and Methods

### Animals

Ten-to 12-wk-old female BALB/c mice were used for immunization and hybridoma generation. For severe combined immunodeficiency-human (SCID-hu) leukocyte migration model, 5-to 6-wk-old female NOD/SCID/IL2r-γ^null^ (NSG) mice were utilized. Both strains were obtained from Jackson Laboratories, Bar Harbor, ME.

*In vivo* experiments were carried out in strict accordance with the recommendations in the Guide for the Care and Use of Laboratory Animals of the National Institutes of Health. The protocols under which these experiments were conducted were approved by VLST’s Institutional Animal Care and Use Committee.

### Human Blood Samples

Human blood was obtained from healthy volunteers in accordance with protocol #20062294, approved by Western Institutional Review Board. Written Informed Consent was obtained for all human subjects participating in this study.

### 18V4F Hybridoma Generation

Ten-to 12-wk-old female BALB/c mice were immunized sequentially with three CC-chemokines in random order: CCL3, CCL4, and CCL5 (PeproTech, Rocky Hill, NJ). For each immunization 10 µg protein was used, following standard immunization protocols. The initial immunizations were carried out with one chemokine in Complete Freund’s Adjuvant (Sigma-Aldrich, St. Louis, MO, #F5881), followed in 3-wk intervals by boosts with each of the two remaining chemokines in Incomplete Freund’s Adjuvant (Sigma-Aldrich, #F5506). Ten d after the final boost, serum was collected and tested for reactivity with the target chemokines by ELISA. Sera were screened at a range of dilutions from 1∶50 to 1∶6400 using biotinylated chemokines (0.5 µg/mL) on streptavidin-coated plates (Thermo Scientific Pierce, Rockford, IL, catalog #15124). Biotinylation of chemokines for ELISA assays was performed using sulfo-NHS-LC-biotin (Thermo Scientific Pierce). Sera incubations were for 90 min at 37°C, plates were blocked using 1% BSA in PBS, and bound antibodies were detected using goat anti-mouse IgG Fc-HRP (Jackson Immuno Research, West Grove, PA, #115-035-071) incubated for 90 min at 37°C. Mice showing significant serum reactivity with the three target chemokines were selected for hybridoma fusions. Mice that showed reactivity with two of the three target chemokines were boosted again with the third chemokine to try to improve antibody responses.

Mice chosen for hybridoma generation were boosted i.p. with a mixture of all three chemokines (20 µg each) in PBS at d-4 and-3 before harvesting the spleens and fusing with NS1 cells (ATCC, Manassas, VA). The fused spleen cells were plated in semi-solid CloneMatrix medium containing fluorescent CloneDetect (Molecular Devices, Sunnyvale, CA), and antibody-secreting clones were picked after 2 wk into 96-well plates using ClonePix FL (Molecular Devices).

Antibodies in the hybridoma supernatant were tested for their ability to recognize CCL3, CCL4, and CCL5 by ELISA (similar to serum tests described above). Cells from fusion wells exhibiting reactivity with multiple chemokines were expanded into 24-well plates for determining the ability to block chemotaxis mediated by the target chemokines. Cells from wells that reacted with the three chemokines and demonstrated at least partial inhibition of chemotaxis were cloned by serial dilution. Clone plate supernatants were again tested by ELISA to identify individual clones that produced antibody that was reactive against multiple chemokines. Clone 18V4F was one of the antibodies confirmed to be multi-reactive. The antibody was purified from culture supernatants using MabSelectSure resin (GE Healthcare Life Sciences, Piscataway, NJ) and determined to be murine isotype IgG2a/λ (IsoStrip, Roche Diagnostics, Mannheim, Germany).

18V4F was confirmed to be specific for CCL3, CCL4, and CCL5 by performing an ELISA against a large panel of chemokines. Chemokines (Peprotech) were directly coated on 96-well plates (Meso Scale Discovery) at 1 µg/mL and probed with 18V4F at 3 µg/mL. As direct coating of the chemokines to ELISA plates may lead to anomalous results due to epitope masking, binding was confirmed using biotinylated chemokines bound to streptavidin plates (Meso Scale Discovery). Bound antibody was detected with SULFO-TAG labeled anti-mouse antibody (Meso Scale Discovery/MSD, Gaithersburg, MD), and the resulting RLU (relative luminescence units) signals were quantitated on a SECTOR Imager 2400 reader (Meso Scale Discovery).

### Humanization of 18V4F

The sequences of the variable domains of 18V4F were determined after PCR amplification of these regions using primers within the constant regions. RNA was extracted from 10^7^ hybridoma cells using RNeasy (Qiagen, Valencia, CA) and reverse transcribed using the SuperScript III Reverse Transcription kit from Invitrogen/Life Technologies (Grand Island, NY). The variable regions were amplified by PCR using a set of primers for either the light chain or the heavy chain [29]. The resulting fragments were inserted into the pCRII plasmid using the Zero Blunt TOPO PCR cloning kit from Invitrogen and sequenced using M13 and M13rev primers.

Humanization of the 18V4F antibody was accomplished using a previously reported approach of CDR grafting into human consensus V_L_ and V_H_ framework sequences [30]. For CDRs H1, H3, L1, and L2 we utilized the structure-based definition of CDR residues, as described by Chothia and Lesk [31], to identify the key residues that may be involved in antigen recognition. For CDRs H2 and L3 we utilized a more inclusive sequence hypervariability-based definition of CDR residues, as described by Kabat *et al.* [32]. Following the Kabat numbering scheme, the 18V4F CDR residues were defined to be the following amino acid stretches: H1 = 26–33, H2 = 52–65, H3 = 95–102; L1 = 25–32; L2 = 50–52; L3 = 91–96. Based on sequence similarity, we chose to use consensus sequences from the V_H_ subgroup III and V_L_ λ subgroup III to serve as a framework for 18V4F CDR residues, while maintaining three murine residues in the V_H_ framework and six murine residues in the V_L_ framework. The sequences representing the C_L_ λ domain and the C_H_1 were precisely joined with the humanized V_L_ and V_H_ sequences, respectively, providing for Fab fragments. The humanized 18V4F Fab was codon-optimized for *E. coli* expression, synthesized by Bio Basic (Ontario, Canada) and cloned into the phage display vector.

### Phage Display Vector

We designed a bicistronic phage vector, pVPD4, for display of Fab fragments containing pelB leader sequences for both the light chain and heavy chain sequences. In the vector, the sequence for the M13 gene III coat protein was fused to the C-terminus of the heavy chain, resulting in display of Fab on the phage surface. The gene III region contained flanking Mlu I restriction digest sites, allowing for excision of the gene III sequence and production of secreted Fab. Following Mlu I digestion, religation introduced a 6-histidine tag to the C-terminus of the heavy chain, providing an affinity tag for downstream expression and purification of Fab fragments.

### Affinity Maturation of 18V4F – Library Construction

Randomized libraries of each CDR were constructed using the method described by Kunkel *et al.* [33]. Specifically, for each CDR library a stop template was generated in which a TAA stop codon was introduced within the CDR sequence to prevent library contamination with wild type sequences. Next, primers were designed to allow randomization of overlapping 5–6 amino acid stretches within the CDR, resulting in 2–3 libraries per CDR with theoretical complexities of 3.2×10^6^–6.4×10^7^. For randomization we used the broadest definition of relevant residues involved in antigen recognition, encompassing both sequence hypervariability-based and structural-based definitions [31], [32]; positions randomized in H1.1 = 26–31, H1.2 = 30–35, H2.1 = 50–55, H2.2 = 56–61, H2.3 = 60–65, H3.1 = 95–100, H3.2 = 98–100c, H3.3 = 100a–102, L1.1 = 24–27b, L1.2 = 27a–30, L1.3 = 29–34, L2.1 = 50–54, L2.2 = 52–56, L3.1 = 89–93, L3.2 = 92–96. Briefly, the phosphorylated primers were annealed to dU-ssDNA templates and enzymatically extended and ligated to form closed-circular DNA as previously described in Sidhu *et al.* [34]. Next, DNA was transformed into electrocompetent *E. coli* SS320 and plated on BioDish XL 2YT-Agar plates containing 50 µg/mL carbenicillin, 5 µg/mL tetracycline, and 1% glucose. The complexity and quality of the resulting libraries were assessed by examining transformation efficiencies and sequencing library clones, respectively. The complexities of the libraries were, for the most part, sufficient to encompass the theoretical complexity of the libraries (H1.1 = 2.3×10^8^, H1.2 = 5.9×10^7^, H2.1 = 2.7×10^8^, H2.2 = 2.3×10^8^, H2.3 = 9.2×10^7^, H3.1 = 1.1×10^8^, H3.2 = 2.2×10^8^, H3.3 = 7.5×10^7^, L1.1 = 4.3×10^7^, L1.2 = 6.0×10^7^, L1.3 = 5.2×10^7^, L2.1 = 7.5×10^5^, L2.2 = 4.5×10^5^, L3.1 = 3.7×10^6^, L3.2 = 1.9×10^6^) with the percentage of template-derived stop codons present in 30–60% of the clones sequenced. Phage particles displaying the humanized 18V4F Fab libraries were made by harvesting the transformed cells and growing in 2YT containing 50 µg/mL carbenicillin, 5 µg/mL tetracycline, and 0.2% glucose. When the culture reached an OD_600_∼0.5, M13K07 helper phage (10^12^ cfu) and kanamycin (10 µg/mL) were added. Following overnight growth at 37°C, phage particles were purified from culture supernatants using two successive PEG-NaCl precipitations. The phage particles were resuspended in 5 mL PBS, titered for concentration determination, and stored at −80°C.

### Affinity Maturation of 18V4F – Screening

For screening, CDR sub-libraries (i.e., H1.1, H1.2) were pooled to yield 6 libraries, each representing a single CDR, and were kept separate throughout the selection process. Prior to each panning experiment, phage particles were pre-depleted on streptavidin magnetic beads (Invitrogen/Life Technologies) in order to reduce non-specific binding. For the first round of selection, ∼10^11^ naïve library phage were added to a solution containing 500 nM of each AviTagged CCL3, CCL4, and CCL5 in 2% casein-PBS. After incubating at 25°C for 1 h, 25 µL of streptavidin magnetic beads (pre-blocked with 2% casein-PBS) were added and allowed to incubate for an additional 30 min. The beads were washed 5–7 times with PBS-0.05% Tween, and bound phage were eluted in 100 mM triethylamine. The resulting eluate was neutralized using 1 M Tris pH 7.5 and infected into XL1 Blue cells for propagation using methods described above. For the following rounds of selection, a similar method was used except the amplified phage were panned against only a single chemokine at a time. Briefly, the second round entailed three separate panning experiments in which ∼10^11^ phage amplified from round one were sequentially panned against 100 nM AviTagged CCL3, CCL4, and CCL5. The bound phage from each sequential panning were washed, eluted and amplified as described above, and individual colonies were sequenced (Sequetech, Mountain View, CA) to monitor sequence enrichment. To obtain affinity matured Fabs, the selection pressure was increased in each round by both lowering the concentration of AviTagged chemokine used in the panning and adding unlabeled chemokine (Peprotech) in 5–10 molar excess to serve as a binding competitor.

### Expression of 18V4F Fab

18V4F Fab variant sequences that were enriched after several rounds of panning were expressed and purified for further characterization. Cleaving the pVPD4 phage vector with Mlu I followed by religation resulted in both the excision of the M13 gene III coat protein and the introduction of a 6-histidine tag to the C-terminus of the C_H_1 domain. T7 Express I^q^ *E. coli* cells containing the Fab expression vectors were grown in shake flasks containing 500 mL 2YT media and 50 µg/mL carbenicillin at 37°C. Upon reaching an OD_600_∼1.0, expression was induced with 0.1 mM IPTG (isopropyl-beta-D-thiogalactopyranoside) and the temperature was shifted to 25–30°C. After 12 h of incubation, the supernatants were harvested, filtered, and subjected to batch-purification with Nickel-IMAC resin (GE Healthcare Life Sciences). Briefly, after overnight incubation with the cleared supernatants at 4°C, the resin was washed with PBS+10 mM imidazole and eluted with PBS+500 mM imidazole. The eluate was dialyzed into PBS and concentrated. A_280_ measurements were used for concentration determination.

### AviTagged Chemokine Expression and Purification

The genes for CCL3, CCL4, and CCL5 were codon optimized for *E. coli* expression, synthesized (Bio Basic) and cloned into the Champion™ pET SUMO expression vector, resulting in a SUMO-chemokine fusion protein containing a 6-histidine tag fused to N-terminus of the SUMO domain and an AviTag-biotinylation sequence (Avidity, Aurora, CO) fused to the C-terminus of the chemokine domain. Single point mutations in the regions associated with chemokine oligomerization were introduced to aid in their expression and solubility, as previously reported (E63S in CCL3, E67S in CCL4, and E66S in CCL5 [35]). RosettaGami™ 2(DE3) cells containing the chemokine expression vectors were grown in shake flasks in TB broth containing 50 µg/mL kanamycin at 37°C. Upon reaching an OD_600_∼0.7, the cells were induced with 1 mM IPTG. After 4 h of incubation at 37°C, the cells were harvested and resuspended in binding buffer (50 mM HEPES pH 7.8, 500 mM NaCl, 100 mM L-arginine, 1% w/v D-mannitol) with the addition of 0.1% Triton X-100, Lysonase (EMD Millipore, Billerica, MA), and protease inhibitors. Cleared lysate was loaded onto a HisTrap-FF column (GE Healthcare Life Sciences), washed with ∼20 column volumes of binding buffer, and eluted using a 0–500 mM imidazole gradient. The SUMO-chemokine containing eluate was buffer exchanged by dialysis into a buffer compatible with BirA biotinylation (50 mM bicine pH 8.3, 10 mM MgOAc, 10 mM ATP, 50 µM D-biotin) and biotinylated with an excess of BirA biotin ligase (Avidity). Following biotinylation, BirA was removed using a HiTrap Q FF ion exchange column (GE Healthcare Life Sciences), and the SUMO domain was enzymatically cleaved by the addition of 6 histidine-tagged ULP1 protease (Invitrogen/Life Technologies). Next, the ULP1 enzyme and SUMO domain, both containing 6-histidine tags, were removed using a HisTrap FF column. The resulting chemokine containing flow through was retained, concentrated, and purified using a size exclusion chromatography column (GE Healthcare Life Sciences) equilibrated in binding buffer.

### Competitive ELISA Measurements

For competitive binding experiments, streptavidin plates (MSD) were coated with biotinylated CCL3 (125 ng/mL). Titrations of the competitors, starting at 2 µM, were added to the plates and incubated at 25°C for 30 min. Next, d5d7 Fab of test antibody was added at 10 µg/mL and incubated at 25°C for 60 min. After washing in PBS-1% Tween, the level of d5d7 Fab bound to the CCL3-coated plate was detected by incubation with a 1 µg/mL SULFO-TAG labeled anti-6-histidine antibody (R&D Systems, Minneapolis, MN).

### Biacore Affinity Determination

Surface plasmon resonance experiments were performed on a Biacore 2000 instrument (GE Healthcare Life Sciences). CCL3, CCL4, and CCL5 (Peprotech) were biotinylated using sulfo-NHS-LC-biotin (Thermo Scientific Pierce) and were immobilized to a streptavidin sensor-chip at a level of ∼100 RUs (resonance units). To measure binding affinities, 18V4F Fab variants were flowed over the chip at varying concentrations (500 pM to 1 µM) followed by regeneration with 10 mM glycine, pH 1.5. All experiments were repeated in triplicate and fit with a 1∶1 Langmuir model, with the exception of the murine and humanized 18V4F Fabs which were fit to a steady state model.

### Generation of MAb d5d7 Full IgG

After several rounds of selection, sequence analysis identified two sequences that were highly enriched: sequence d5 within CDR-L1 and sequence d7 within CDR-H1. These sequences were combined to generate MAb d5d7. The variable domain of the 18V4F_d7 heavy chain was subcloned in frame with human IgG4 Fc domain containing the S228P stabilization mutation [36] and expressed from the elongation factor promoter in vector pNEF50 [37]. The 18V4F_d5 light chain was subcloned into pHLEF50 vector [37]. The expression vectors were co-transfected into 293-EBNA cells, and antibodies in the supernatant were purified using MabSelectSure (GE Healthcare Life Sciences) followed by a Superdex200 size exclusion column (GE Healthcare Life Sciences).

**Figure 1:**
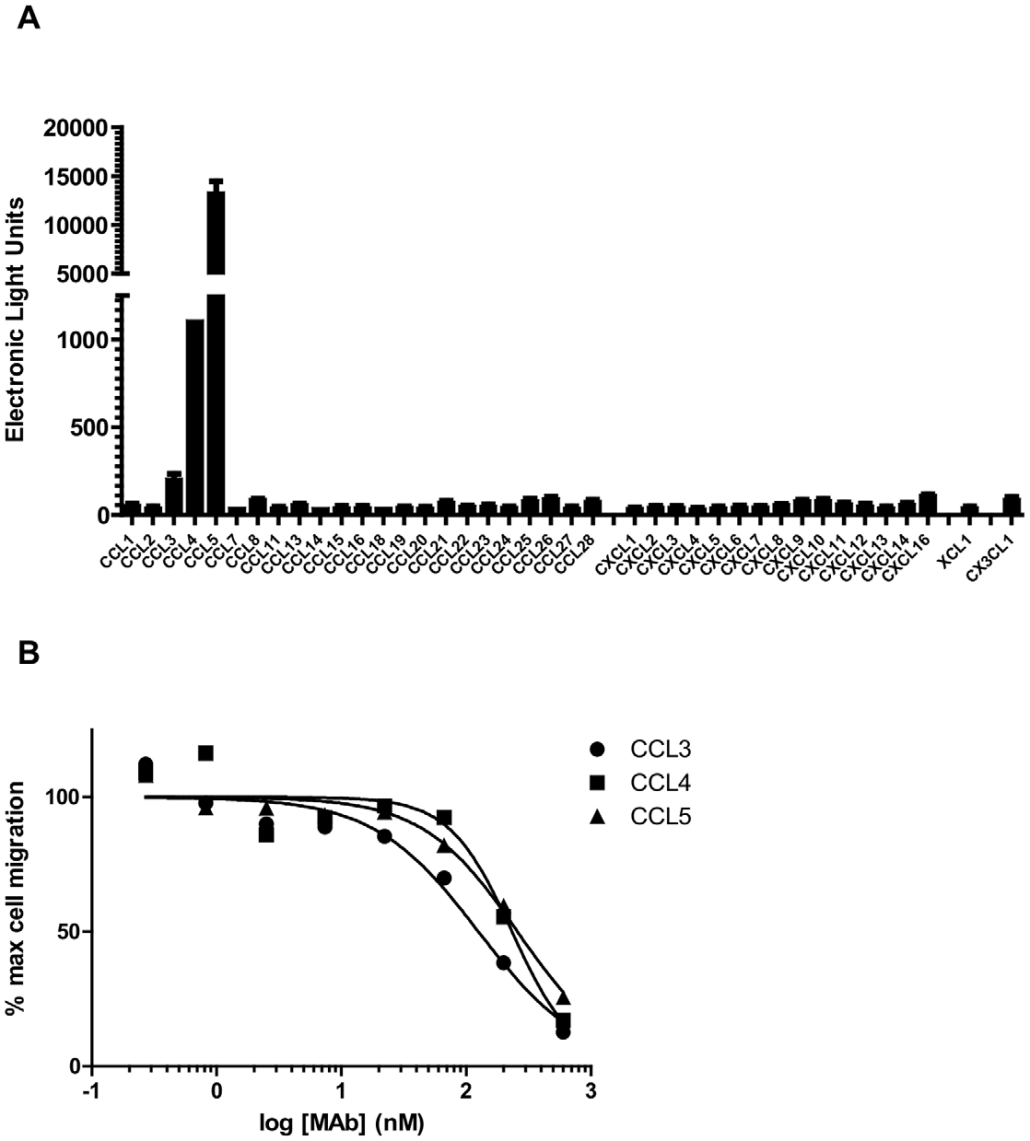
Binding and *in vitro* activity of murine 18V4F hybridoma antibody. (**A**) ELISA binding of original 18V4F hybridoma antibody to a panel of chemokines (determined in triplicate, shown as mean +/− standard deviation). Directly coated chemokines were used here for direct comparisons, however in other experiments CCL3 showed a significantly enhanced signal when biotinylated and coated on streptavidin plates. (**B**) Chemotaxis inhibition by 18V4F hybridoma antibody of CCR5-transfected Ba/F3 cells to 5 ng/mL of CCL3, CCL4, and CCL5. Data are representative of at least three similar experiments. All chemotaxis data are represented as a percent of maximum migration in the absence of inhibitors and is fit using a standard four parameter dose-response model (GraphPad).

### Chemotaxis

Chemotaxis assays were performed in 96-well transwell plates (Corning, Corning, NY, catalog #3388) using CCR5 or CCR1 transfectants. CCR5 and CCR1 expression vectors were made by amplifying the open reading frames from cDNA clones (Origene, Rockville, MD) and inserting into pNEF38 [37]. Ba/F3 cells (DSMZ, Braunschweig, Germany) were transfected with the resulting plasmids by electroporation (Amaxa/Lonza, Allendale, NJ) and selected in G418. Cells expressing functional receptor were selected through chemotaxis to the cognate ligands, and migrating cells were cloned by limiting dilution to obtain a stable high-expressing cell clone. Chemotaxis assays were performed in RPMI with 2% FBS using 3×10^5^ transfectants per well. Chemokines (from Peprotech) with or without inhibitors (MAb d5d7 antibody or controls) were added into the lower chambers. Migrating cells were harvested after 2 h and quantitated using CellTiter-Glo Luminescent Cell Viability Assay (Promega, Madison, WI). Control inhibitors included vCCI-Fc (produced at VLST – the Fc is from human IgG1 with mutations to prevent interactions with Fc receptors [38]) and commercial antibodies against CCL3 (R&D Systems #MAB270), CCL4 (R&D Systems #MAB271), and CCL5 (R&D Systems #MAB278) as well as IgG controls. Concentrations of chemokines used to induce chemotaxis are specified in the figure legends for each experiment and ranged from 3–20 ng/mL depending on the chemokine and the specific experimental conditions tested. The selected concentrations produced 50% maximal chemotaxis. Most assays used chemokines from Peprotech to induce chemotaxis, with the exception of experiments that used a supernatant containing inflammatory chemokines from LPS (lipopolysaccharide)-stimulated PBMC (peripheral blood mononuclear cells). This supernatant was generated by stimulation of human PBMC at 10×10^6^ cells/mL with LPS (Sigma) at 1 µg/mL and recombinant human IFNγ (Peprotech) at 1000 U/mL in the presence of 50 U/mL IL-2 (Peprotech) for 48 h. The levels of CCL3, CCL4, and CCL5 in the supernatant were quantitated by Rules Based Medicine (Austin, TX) and found to be at 71 ng/mL, 398 ng/mL, and 1.7 ng/mL, respectively. Chemotaxis results are expressed as percent of the maximum number of cells migrating into the lower chamber induced by chemokine without inhibitor present.

**Table 1:** Affinity and potency measurements of 18V4F Fab variants.

Fab	CDR-L1*	CDR-H1*	ligand	*k*_a_×10^5^(s^−1^ M^−1^)	*k*_d_×10^−3^(s^−1^)	*K*_D_ (nM)	IC_50_ (nM)
**Mu-18V4F**	RSNTGAVTTSNYAN	GFSLTGYGIH	CCL3	–	–	208.7+/−92.4	196+/−134
			CCL4	–	–	103.3+/−25.8	185+/−95
			CCL5	–	–	50.3+/−27.8	>1000
**Hu-18V4F**	SSNTGAVTTSNYAN	GFSLTGYAMH	CCL3	–	–	271.7+/−112	>1000
			CCL4	–	–	251.0+/−63.4	>1000
			CCL5	–	–	187.3+/−71.2	>1000
**d5**	SSNTPLRPYRNYAN	GFSLTGYAMH	CCL3	1.0	3.1	30.4+/−11.7	187+/−190
			CCL4	0.7	1.2	15.9+/−3.7	49+/−67
			CCL5	3.4	1.4	5.5+/−3.4	105+/−48
**d7**	SSNTGAVTTSNYAN	GFSLYDEGAH	CCL3	1.1	3.3	35.0+/−16.0	18+/−5
			CCL4	0.6	0.4	7.3+/−0.2	0.6+/−0.6
			CCL5	2.3	0.1	5.5+/−0.6	4.1+/−0.1
**d5d7**	SSNTPLRPYRNYAN	GFSLYDEGAH	CCL3	0.8	5.4	8.5+/−3.5	1.0+/−0.6
			CCL4	3.8	3.6	1.8+/−1.3	0.2+/−0.1
			CCL5	9.6	0.1	1.6+/−0.6	0.9+/−0.4

* Underlined positions indicate mutations that were found to be enriched throughout the selection process. Affinity measurements were obtained as described in the Methods section. Standard deviations are reported and calculated from at least three independent experiments.

### CCR5^Ser349^ Phosphorylation Assay

Chemokine-dependent phosphorylation of T cell CCR5^Ser349^ in human PBMC was determined by phosphoflow cytometry, as previously described with whole blood samples [39]. Briefly, 1×10^6^ PBMC in 50 µL were incubated with or without inhibitors (MAb d5d7 antibody or controls) for 30 min at 37°C followed by incubation with a mixture of CCL3, CCL4, and CCL5 (50 ng/mL each) for 15 min. Samples were incubated in lyse/fix buffer (BD Biosciences) and pelleted twice, then washed and stored frozen overnight in 100% methanol. Samples were then stained with PE-labeled anti-CCR5^pSer349^ (Biolegend, San Diego, CA, clone E11/19) plus FITC-CD8 and PerCP-CD4 (BD Biosciences, San Diego, CA) and analyzed on a FACSCalibur flow cytometer. Results are expressed as percent of the maximum number of CCR5^pSer349^-positive CD8 T cells after chemokine induction without inhibitor present. Experiments demonstrating MAb d5d7 inhibition were confirmed with blood samples from six different donors.

**Figure 2:**
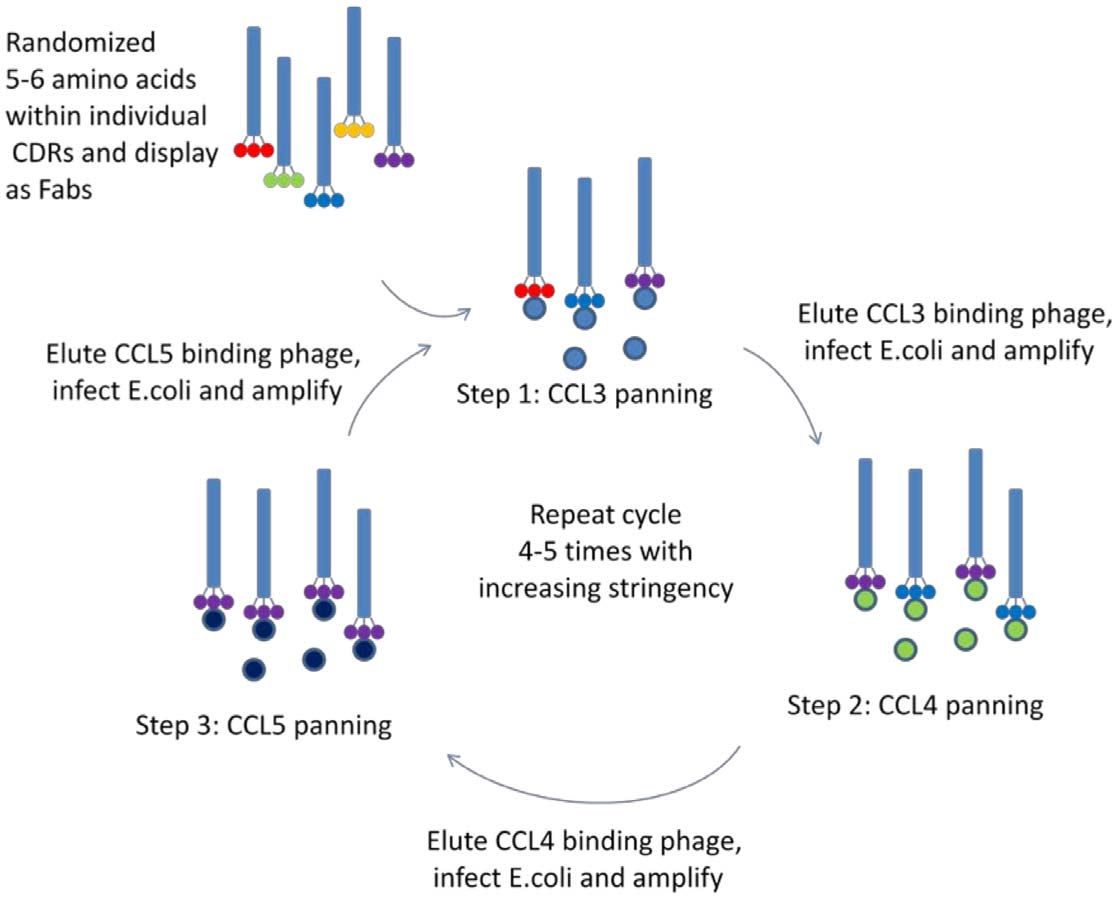
Diagram of phage display selection strategy. Individual CDR libraries were sequentially panned against CCL3, CCL4 and CCL5. In step 1, phage libraries were combined with biotinylated CCL3 and bound to streptavidin beads. Bound phage were eluted, amplified, and subjected to panning against biotinylated CCL4 and CCL5 in steps 2 and 3, respectively. This process was repeated 4–5 times with increasing stringency to yield sequences with improved affinities.

### CD11b up-regulation Assay

Chemokine-induced CD11b up-regulation on monocytes was assayed in whole human blood as previously described [40]. Briefly, 20 µL blood samples were incubated with or without inhibitor (MAb d5d7 antibody or controls) for 5 min at 37°C followed by incubation with a mixture of CCL3 and CCL5 (16 ng/mL and 80 ng/mL, respectively) for 15 min. Reactions were stopped on ice and azide was added. CD11b levels on monocytes were determined by measuring the mean fluorescence intensity of Bear-1 antibody binding (FITC labeled, Santa Cruz Biotechnology, Santa Cruz, CA) on a FACSCalibur flow cytometer. Results are expressed as percent of the maximal increase in mean fluorescence intensity determined after chemokine induction without inhibitor present. Experiments demonstrating MAb d5d7 inhibition were confirmed with blood samples from eight different donors.

**Figure 3:**
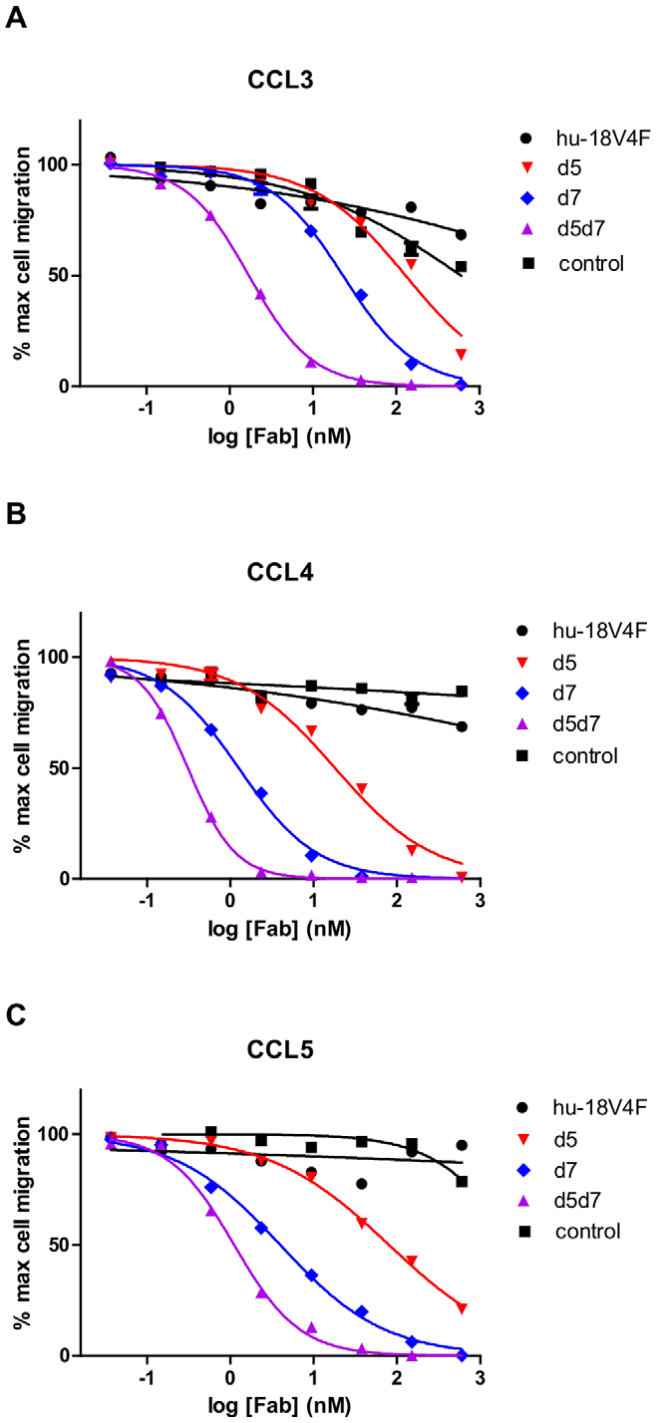
Chemotaxis inhibition by affinity matured 18V4F variants. Chemotaxis inhibition by humanized 18V4F Fab, d5 variant, d7 variant, d5d7, and a negative control Fab of CCR5-transfected Ba/F3 cells to 5 ng/mL of (**A**) CCL3, (**B**) CCL4, and (**C**) CCL5. Data are representative of at least two similar experiments. A loss in potency of humanized 18V4F Fab was observed compared with the 18V4F hybridoma shown in Figure 1b and is likely a result of both the humanization process and loss in avidity caused by switching from full IgG to Fab fragment.

### *In vivo* Skin Leukocyte Migration Model

A SCID-hu mouse skin leukocyte migration model was developed to test the ability to inhibit human chemokine function *in vivo*. Five-to 6-wk-old female NSG mice were injected i.v. with 1.5×10^7^ human PBMC per mouse. Human cells represented ∼30% of blood leukocytes at the end of the experiment (d 17). On d 10, Matrigel (BD Biosciences) plugs containing chemokines (or PBS) were injected subcutaneously on the abdomen of mice, two plugs per mouse. A combination of 0.4 µg CCL3, 0.4 µg CCL4, and 0.4 µg CCL5 were mixed into each 50 µL Matrigel sample. Also on d 10, just prior to chemokine injections, and again on d 13 and 15, 500 µg inhibitor (MAb d5d7 antibody or controls) in 200 µL PBS (or PBS alone) was administered intravenously. On d 17, mice were sacrificed, abdominal skin was removed, and skin samples containing Matrigel plugs were harvested with a 6 mm punch biopsy tool. These samples were digested in 0.5 mL dispase (Invitrogen)/DNase (Roche) (1.2 U/mL and 25 µg/mL, respectively) for 1 h at 37°C and pressed through 70 mm nylon mesh. Resulting individual cell suspensions were stained for human leukocyte cell surface markers and analyzed on a FACSCalibur flow cytometer. Results are expressed as the percentage of human CD3-positive cells in the digested skin sample. Inhibition by MAb d5d7 was demonstrated in five separate experiments.

**Figure 4:**
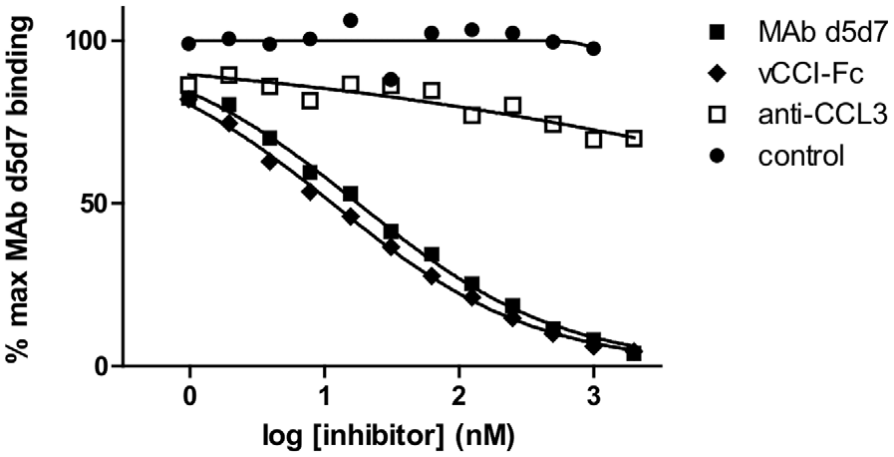
Comparison of vCCI and d5d7 binding epitopes. Competitive binding ELISA examining molecules that can disrupt the d5d7-CCL3 binding interaction using d5d7 as a homologous competitor and vCCI-Fc, commercial anti-CCL3 antibody, and control IgG as heterologous competitors. Data are representative of at least two similar experiments. Competition experiments were also completed to analyze the d5d7-CCL4 and d5d7-CCL5 binding interactions and similar binding competition was observed between d5d7 and vCCI-Fc (data not shown).

## Results

### Generation of a Multi-chemokine Specific Antibody

While CCL3, CCL4, and CCL5 share a similar tertiary chemokine fold, the sequence identity amongst all three chemokines is quite low (∼40%) and is interspersed throughout the primary sequence with the longest contiguous stretch of identity being only four amino acids long. As such, we expected antibodies that displayed cross-reactivity with CCL3, CCL4, and CCL5 to target a complex tertiary epitope and to be relatively rare. Therefore, we utilized a sequential immunization strategy that would amplify antibody responses to conserved epitopes within the three chemokines as opposed to a method that involved simultaneous immunization with all three chemokines. Mice were immunized with CCL3, CCL4, and CCL5 sequentially in all combinations of order. Sera from immunized mice were assessed by ELISA, and those with highest titers against all three chemokines were queued for hybridoma generation.

**Figure 5:**
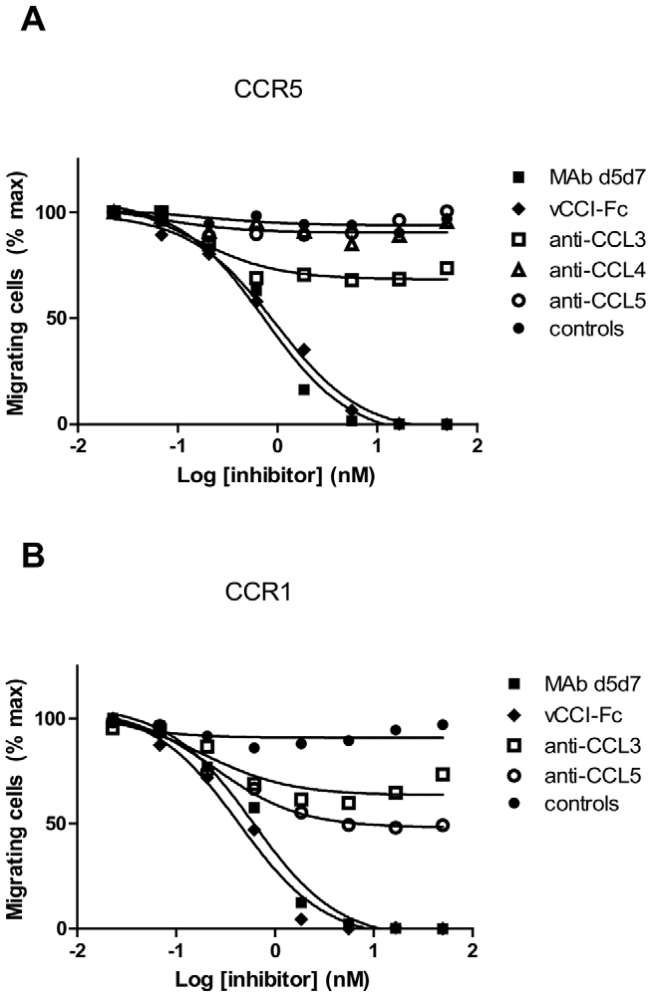
Inhibition of chemotaxis induced with mixtures of chemokines by MAb d5d7. Inhibition of chemotaxis of (**A**) CCR5 transfectants to a pool of recombinant CCL3, CCL4, and CCL5 and (**B**) CCR1 transfectants to a pool of CCL3 and CCL5, by MAb d5d7 antibody, vCCI-Fc, individual commercial anti-chemokine antibodies (anti-CCL3, anti-CCL4, and anti-CCL5), and IgG controls. Chosen chemokine concentrations were those that produced 50% maximal chemotaxis when tested individually (a pool of 3 ng/mL CCL3, 10 ng/mL CCL4, and 3 ng/mL CCL5 was used in CCR5 experiments and a mixture of 20 ng/mL CCL3 and 5 ng/mL CCL5 was used in CCR1 experiments).

A total of 18 spleens from immunized mice were used for hybridoma fusions to identify multi-reactive monoclonal antibodies. Tens of thousands of hybridoma clone supernatants were screened in order to identify monoclonal antibodies with our desired characteristics. The primary screen for identifying triple-reactive antibodies was an ELISA-based assay examining binding of secreted antibodies contained in hybridoma supernatants to CCL3, CCL4, and CCL5. Positives were checked for clonality to ensure identification of triple-reactive monoclonal antibodies and not mixtures of multiple antibodies with single or double-reactivities. Once triple-reactive antibodies were confirmed, a secondary ELISA-based screening assay assessed the antibodies’ profiles of binding to a panel of 40 commercially available chemokines, confirming reactivity with only the three targeted chemokines CCL3, CCL4, and CCL5. Lastly, these antibodies were tested for their ability to inhibit chemotaxis of CCR5-transfected cells induced with CCL3, CCL4, and CCL5. CCR5-tranfected cells were chosen for this assay as they respond to all three chemokines, unlike CCR1 transfectants, and display a high level of reproducibility compared with native leukocytes. A single antibody, 18V4F, was found to bind CCL3, CCL4, and CCL5 in the primary ELISA screen (Figure 1a) and inhibit their chemotactic functions with IC_50_ values of 190–1000 nM (Figure 1b and Table 1). Additional characterization of the binding affinity using Biacore indicated that 18V4F bound to CCL3, CCL4, and CCL5 with K_D_ values of 208.7 nM, 103.3 nM, and 50.3 nM, respectively (Table 1).

**Table 2:** IC_50_ measurements of MAb d5d7 and vCCI with different cell types.

		95% confidence intervals for IC_50_ calculations (nM)					
Assay	chemokine	MAb d5d7			vCCI		
		CCR1^+^	CCR5^+^	PBMC	CCR1^+^	CCR5^+^	PBMC
**Chemotaxis**	CCL3, (4)*, 5	0.4–0.9	0.5–1.0	–	0.3–0.6	0.8–1.2	–
	PBMC CM#	0.6–0.7	0.5–0.9	–	2.5–3.9	0.3–0.4	–
**CCR5^pSer349^**	CCL3, 4, 5	–	–	2.7–6.7	–	–	11.1–25.5
**CD11b**	CCL3, 5	–	–	6.7–10.9	–	–	12.4–20.2

CCR1^+^ and CCR5^+^ refer to Ba/F3 cells that have been transfected with these receptors.

–Indicates value was not measured.

* Denotes CCL4 was included when assaying CCR5 expressing cells.

# CM denotes conditioned medium.

### Humanization and Phage Display-based Affinity Maturation Improves Affinity against CCL3, CCL4, and CCL5

The antibody variable domains of 18V4F were humanized using a consensus framework approach similar to that previously employed [30], resulting in some loss in affinity towards its ligands (Table 1). While both the original murine and the humanized 18V4F antibodies contained the desired binding specificity, both the affinity and *in vitro* potency were weaker than that desired of therapeutic antibodies (typical K_D_ values range from 0.1–10 nM [41]). Therefore, affinity maturation using phage display was carried out in order to improve binding affinities and *in vitro* potency of 18V4F against CCL3, CCL4, and CCL5. Fab libraries based on the humanized 18V4F sequence were constructed in which 5–6 amino acid stretches within the complementarity determining regions (CDRs) were randomized and displayed on the surface of M13 phage.

**Figure 6:**
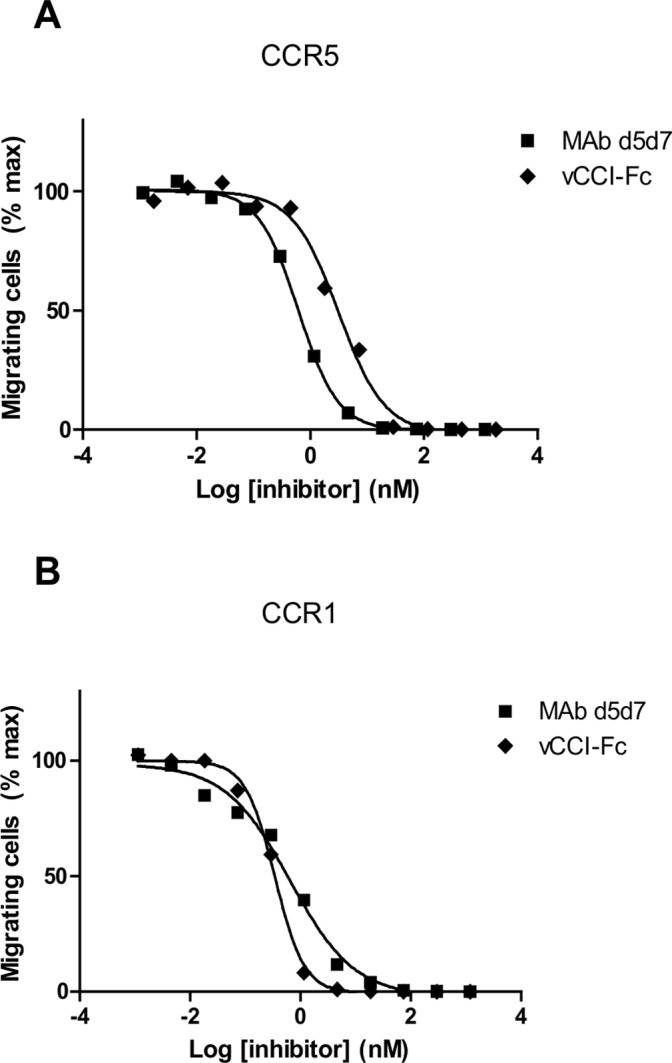
Inhibition of chemotaxis induced with native chemokines by MAb d5d7. Chemotaxis inhibition by MAb d5d7 antibody and vCCI-Fc of (**A**) CCR5 transfectants and (**B**) CCR1 transfectants to a supernatant containing inflammatory chemokines from LPS-stimulated PBMC. The dilution of supernatant used in the assay was that which produced 50% maximal chemotaxis (1∶80 for CCR5 cells and 1∶20 for CCR1 cells). Data are representative of at least two similar experiments.

As most affinity maturation efforts are focused on improving affinity to a single ligand, we developed a novel selection strategy involving sequential panning against biotinylated CCL3, CCL4, and CCL5 to explicitly select for improved binding to all three ligands (Figure 2). Since a single round of selection would require sequential binding to all three chemokines, we anticipated that improvement to only one or two chemokines would be unlikely and our desired breadth of binding would be maintained. Additionally, as the panning rounds progressed, standard methods for increasing stringency were used, including decreasing concentrations of biotinylated chemokines used in panning and adding excess unlabeled chemokines to select for sequences with reduced off-rates [42], [43]. After several rounds of selection, sequence analysis identified two sequences that were highly enriched: sequence d5 within CDR-L1 and sequence d7 within CDR-H1 (Table 1).

**Figure 7:**
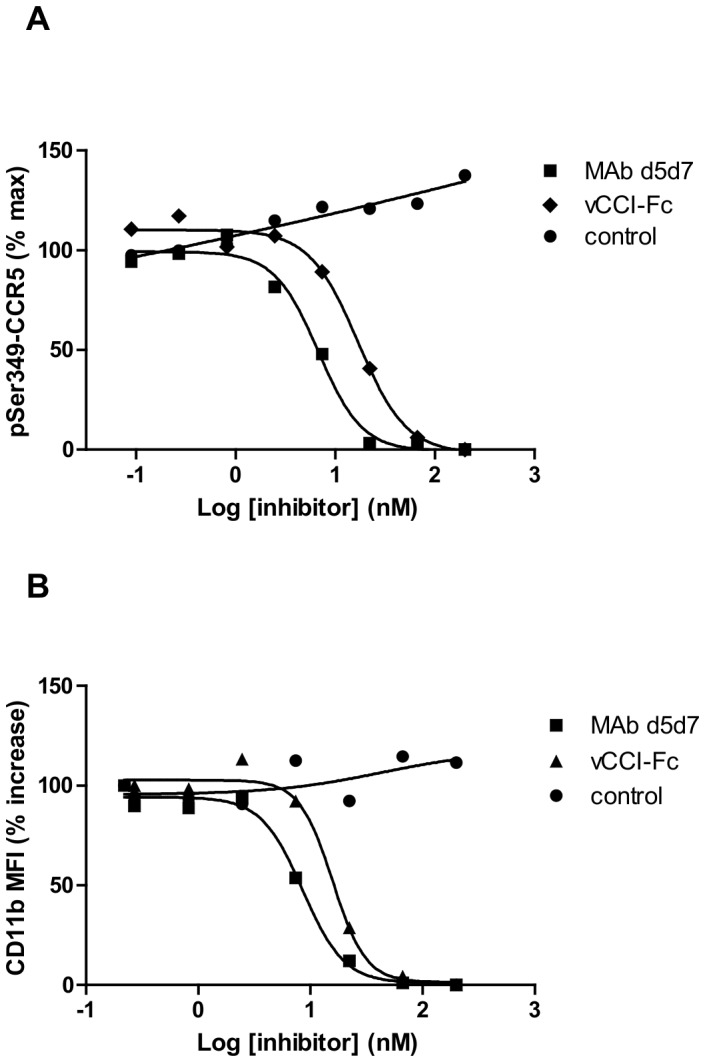
Inhibition of chemokine signaling on native leukocytes by MAb d5d7. (**A**) Inhibition by MAb d5d7 antibody, vCCI-Fc, and IgG controls of chemokine-induced phosphorylation of CCR5^Ser349^ in CD8+ T cells. Phosphorylation was induced with a pool of 50 ng/mL each CCL3, CCL4, and CCL5 (each of which produced ∼80% maximal response when tested individually). Data are expressed as percent of the maximum number of CCR5^pSer349^-positive CD8+ T cells after chemokine induction without inhibitor present and are representative of results using PBMC from six different donors. (**B**) Inhibition of CD11b up-regulation on monocytes induced with a pool of CCL3 and CCL5. Chosen chemokine concentrations were those that produced ∼80% maximal response when tested individually (16 ng/mL CCL3 and 80 ng/mL CCL5). Data are expressed as percent of the maximal increase in mean fluorescence intensity determined after chemokine induction without inhibitor present and are representative of results using blood samples from eight different donors.

To characterize the effects of these sequence variations, soluble Fabs were generated containing the d5 and d7 mutations individually and in combination (d5d7). Biacore analysis indicated a ∼10-to ∼30-fold improvement in binding affinities against CCL3, CCL4, and CCL5 for the individual d5 and d7 sequences compared with the humanized 18V4F (Table 1). Importantly, these improvements were more than additive when both mutations were present, resulting in up to a ∼100-fold enhancement in affinity of d5d7 for CCL3, CCL4, and CCL5 compared with humanized 18V4F (Table 1). ELISA assays testing the binding of d5d7 against a broad array of chemokines (similar to Figure 1a) indicated that, while the binding affinity of d5d7 to CCL3, CCL4, and CCL5 was improved, the overall binding profile remained the same (data not shown).

**Figure 8:**
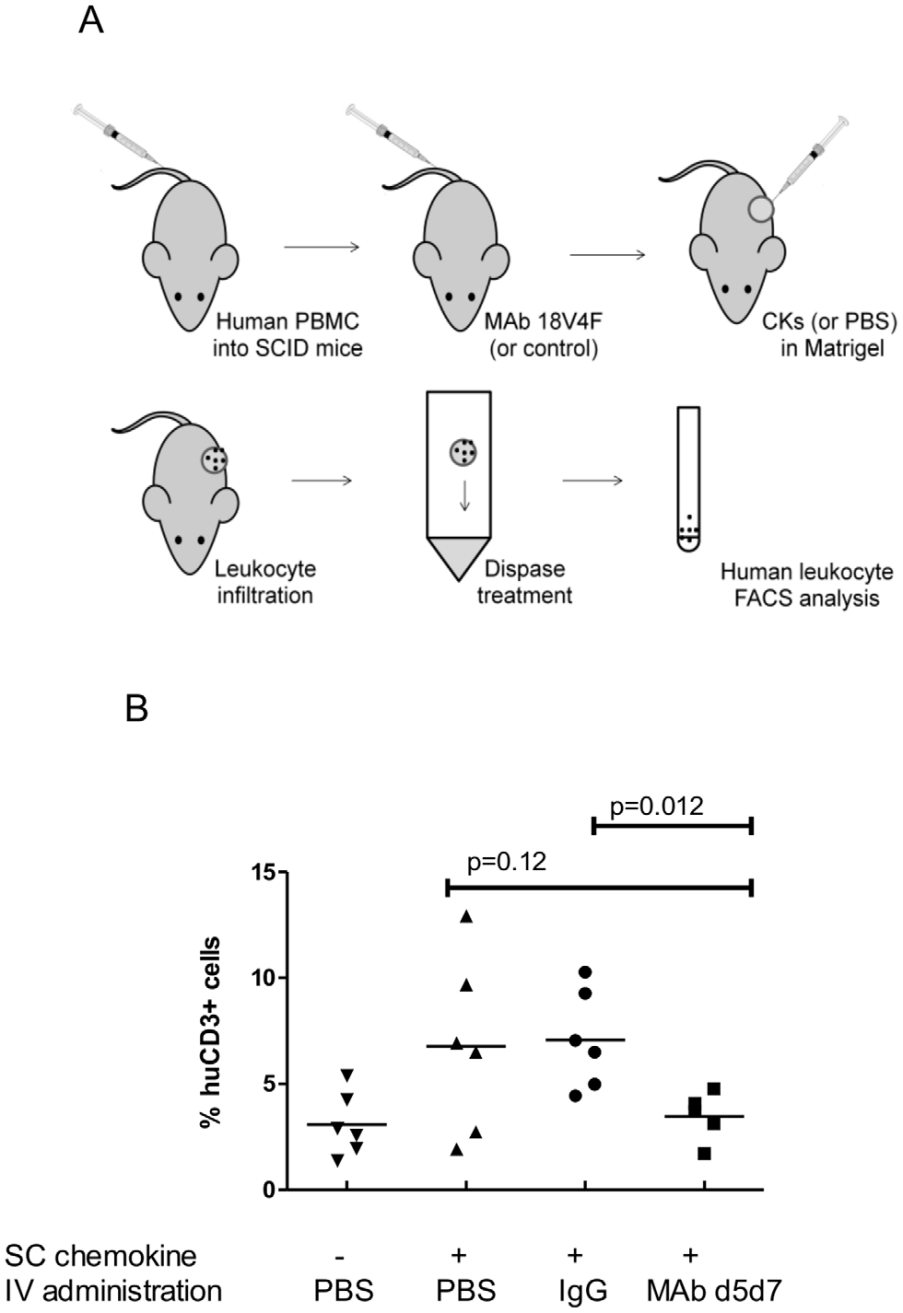
SCID-hu mouse model of leukocyte migration. (**A**) NSG (NOD/SCID/IL2r-γnull) mice were injected i.v. with human PBMC and allowed to engraft for 10 d. MAb d5d7 was administered i.v. just before chemokines were injected s.c. in Matrigel. After 7 d the skin sites were harvested and single cell suspensions were generated. Human leukocytes were tagged with specific antibodies and analyzed by flow cytometry. (**B**) Inhibition by MAb d5d7 of skin leukocyte migration into chemokine-embedded Matrigel plugs in NSG mice engrafted with human PBMC. The negative control group consisted of animals treated with s.c. injection of Matrigel + PBS and i.v. administration of control IgG. All other groups had s.c. injections of Matrigel containing CCL3, CCL4, and CCL5 (400 ng each) with i.v. administration of PBS, control IgG, or MAb d5d7 antibody. Data were analyzed using a student t test.

Given the observed enhancement in affinity of the d5 and d7 mutations, both individually and in combination, we examined whether this improvement translated to increased potency in chemotaxis inhibition. The d5 and d7 individual variants displayed improved *in vitro* potency in inhibiting chemokine-induced migration of CCR5 expressing cells (Figure 3). Both mutations in combination, d5d7, resulted in a greater than 1000-fold enhancement of inhibition of CCL3, CCL4, and CCL5-induced chemotaxis compared with the humanized 18V4F Fab (Figure 3 and Table 1). Thus, our selection strategy was successful at identifying sequences that resulted in simultaneous improvement in both the affinity and *in vitro* potency of 18V4F towards CCL3, CCL4, and CCL5.

While neither the antibody generation nor the affinity maturation experiments were designed to yield an antibody targeting the same binding site employed by vCCI, binding competition experiments indicate that the binding regions of d5d7 and vCCI overlap (Figure 4). This suggests the presence of a conserved region on the surface of chemokines that allows for their broad neutralization. As chemokines are relatively small proteins, one might expect a limited number of non-overlapping epitopes that display neutralizing activity. Thus, it is interesting to note that, while the binding site of d5d7 overlaps with that of vCCI, it does not appear to overlap with that of a highly potent commercial anti-CCL3 antibody (Figure 4).

### MAb d5d7 is a Potent Inhibitor of Recombinant Chemokines and Native Chemokines Produced by PBMC

For further experimentation confirming the potent activity of d5d7 Fab, the Fab was converted to full IgG format (termed MAb d5d7). Consistent with the d5d7 Fab data, MAb d5d7 potently inhibits CCR5-mediated chemotaxis induced by individual chemokines (data not shown). Importantly, if MAb d5d7 functions as a multi-chemokine inhibitor, we would expect to observe similar chemotactic inhibition with MAb d5d7 when a mixture of relevant chemokines is used to induce chemotaxis as opposed to individual chemokines. Figure 5a indicates that MAb d5d7 inhibits chemotaxis of CCR5-transfectants induced by a mixture of recombinant CCL3, CCL4, and CCL5 with a potency equivalent to that of vCCI (Table 2). Similarly, MAb d5d7 inhibits chemotaxis of CCR1-transfectants to a mixture of CCL3 and CCL5, the primary ligands for CCR1 (Figure 5b, Table 2). Not surprisingly, commercial antibodies with high potency against single chemokines were ineffective at inhibiting chemotaxis induced by this chemokine mixture.

While we have demonstrated that MAb d5d7 is capable of potently inhibiting recombinant chemokines either individually or in combination, sites of inflammation in an *in vivo* setting contain a much more complex mixture of chemokines. For example, not only may additional chemokine ligands for the CCR1 and CCR5 receptors be present, such as CCL15 and CCL23, but additional chemokine isoforms may also be present. These isoforms may exist due to expression from related genes (such as CCL3 and CCL3L1) or from proteolytic processing (such as removal of amino-terminal residues of CCL3 by CD26) [44], [45] and may be more potent ligands for CCR1 and CCR5 than the three primary ligands tested. To approximate these more complex native conditions, we prepared conditioned media from PBMC stimulated with LPS and IFNγ, a combination known to induce a broad spectrum of chemokines from human PBMC. Using this conditioned media to induce chemotaxis of CCR1 and CCR5 transfected cells, Figure 6 demonstrates that MAb d5d7 inhibits chemotaxis with a similar potency as vCC1 (Table 2). These data suggest that MAb d5d7 effectively inhibits the native ligands for CCR1 and CCR5 which are produced by activated human PBMC under these conditions, and that an antibody inhibiting the three target chemokines is equally effective as vCCI which targets the entire spectrum of CC chemokines.

### MAb d5d7 Blocks CCL3, CCL4, and CCL5-induced Activation of Native Cell Populations through CCR1 and CCR5

To evaluate the ability of MAb d5d7 to inhibit chemokine signaling in primary leukocytes rather than cells engineered to express the chemokine receptors, we developed signaling assays to assess a number of chemokine-dependent downstream signaling events using human leukocytes *ex vivo*. Chemokine binding to chemokine receptors triggers a cascade of events that eventually lead to cell migration and/or activation. These may include receptor phosphorylation, G protein activation, MAPK activation, and integrin up-regulation. We utilized an assay with primary leukocytes *ex vivo* to measure chemokine-induced phosphorylation of CCR5^Ser349^ on T cells, which reflects an early signaling event in chemokine-induced cell responses. This assay has been used to demonstrate efficacy of CCR5 inhibitors in the clinic [39]. The phosphorylation of CCR5 induced by a mixture of CCL3, CCL4, and CCL5 (all potent CCR5 ligands) was fully inhibited by MAb d5d7 and vCCI in a dose-dependent manner (Figure 7a, Table 2).

Another assay monitoring the level of chemokine-induced CD11b integrin up-regulation on monocytes examines a key downstream event after CCR1 stimulation, which is necessary for chemokine-induced cell extravasation and migration. This activity induced by a mixture of CCL3 and CCL5 was also found to be inhibited by both MAb d5d7 and vCCI (Figure 7b, Table 2). Additionally, both vCCI and MAb d5d7 were shown to inhibit chemotaxis of PBMC induced by both CCL3 and CCL5 (data not shown). Results of these assays extend the observation that MAb d5d7 effectively inhibits chemokine-mediated chemotaxis and provide evidence that antibody binding of targeted chemokines prevents receptor engagement and subsequent triggering of chemokine receptor signaling events in native leukocytes as well as receptor transfectants.

### Administration of MAb d5d7 Inhibits Leukocyte Migration in a SCID-hu Mouse Model

The inhibitory activity of MAb d5d7 was also demonstrated in a novel *in vivo* model of chemokine-induced inflammation. The antibody did not show significant cross-reactivity to rodent chemokines, so traditional mouse inflammatory disease models could not be utilized. Therefore, to evaluate the ability of MAb d5d7 to inhibit cell migration in a representative *in vivo* setting, a novel skin leukocyte migration model was developed to simulate a local inflammatory environment (Figure 8a). This model merged components of a number of previously described tests of both chemokine and growth factor inhibitors [46]–[48]. In this model, immune-compromised SCID-hu mice engrafted with human PBMC were injected subcutaneously with human chemokines. This system allows for interactions between human leukocytes and human chemokines in the *in vivo* context of the SCID-hu mouse, establishing the setting for the complex migration of leukocytes through the endothelial layer into the tissue along a chemokine gradient. The human chemokines were injected subcutaneously in a Matrigel matrix to induce a focal site of inflammation in the skin of the engrafted SCID-hu mice. After 7 d, migration of human leukocytes into the area was analyzed by flow cytometry and the ability of MAb d5d7 to block cell migration into the tissue environment was assessed. Systemic administration of MAb d5d7 completely inhibited engrafted human T cell infiltration into the skin induced by a combination of human CCL3, CCL4, and CCL5 in this inflammation model (Figure 8b). These results demonstrate that the chemokine-neutralizing activity of MAb d5d7 observed *in vitro* translates to the more complex environment *in vivo* where leukocytes must navigate out of the circulatory system through the endothelium and into tissue sites of inflammation.

## Discussion

Chemokines and chemokine receptors have been implicated in pathogenic mechanisms of numerous inflammatory disorders; thus, development of therapeutics aimed at antagonizing their activity is of much interest. Here, we describe the generation and characterization of an antibody that is capable of binding and inhibiting the functions of CCL3, CCL4, and CCL5, prominent chemokines orchestrating leukocyte migration during inflammation via the CCR1 and CCR5 receptors. Through a combined sequential immunization and affinity maturation strategy we obtained a cross-reactive antibody with equivalently high affinities and inhibitory functions towards three proinflammatory chemokines, CCL3, CCL4, and CCL5. Initial studies have shown that MAb d5d7 potently inhibits chemotaxis of both CCR1-and CCR5-transfected cells induced with recombinant CCL3, CCL4, and CCL5. Importantly, this antibody also potently inhibits native chemokines found in conditioned media from stimulated PBMC, likely more representative of the complex, native inflammatory environment containing a broader number of chemokine ligands and isoforms. Additional studies indicate that MAb d5d7 is also capable of inhibiting chemokine-induced signaling events, CD11b up-regulation and CCR5 phosphorylation, on native leukocytes. Lastly, MAb d5d7 blocks leukocyte migration in an *in vivo* setting as demonstrated in a novel SCID-hu skin leukocyte migration model.

Several areas of research have provided evidence for the relevance of CCR1, CCR5, and their primary ligands in disease pathogenesis for both RA and MS. Along with enhanced expression at the sites of inflammation in RA [3], [6]–[8] and MS [9]–[13], both receptors and their ligands have been shown to be important in rodent models of disease. Specifically, the absence of CCR1 signaling via small molecule inhibition or gene deletion had ameliorative effects in CIA models of RA and EAE models of MS [17], [49]. Similarly, in CIA models, blockade of CCR5 signaling with a small molecule inhibitor reduced both the incidence and severity of disease [20]. However, CCR5-deficient mice displayed no protection against development of EAE [19]. In addition, there is evidence that the CCR5Δ32 mutation in humans, which results in loss of function of CCR5, is modestly protective against RA [50]–[52] and correlates with prolonged disease-free intervals in MS patients [53], [54]; however, these findings have not been consistent [55]–[58]. Efforts to inhibit the function of CCL3 and CCL5 have also shown improved outcomes in EAE and CIA disease models [15], [16], [18].

Despite promising results in animal models, to date, there have been no therapeutic drugs targeting chemokines or chemokine receptors approved for the treatment of autoimmune diseases (see [21] for review). A small molecule inhibitor of CCR5, Maraviroc, has been approved for treatment of HIV but failed to demonstrate efficacy in the treatment of RA patients [59]. More recently, promising but preliminary results were reported for the CCR1 inhibitor CCX354-C in RA patients as well as the CCR9 inhibitor CCX282 in Crohn’s disease and inflammatory bowel disease [60]. Several explanations for the limited success have been posited, including insufficient coverage of small molecule chemokine receptor antagonists [22], as well as functional redundancy in the chemokine signaling system [21]. Due to this redundancy, it has been proposed that a therapeutic targeting chemokine signaling may be required to recognize and block several chemokines and/or chemokine receptors in order to effectively ameliorate disease [61], [62]. Strategies to tackle redundancy have involved design of therapeutics based on modification of chemokine ligands (metRANTES; [63], [64]) allowing for simultaneous blockade of CCR1 and CCR5, as well as therapeutics based on soluble chemokine receptors (soluble CCR5-Ig; [65]) to bind and inhibit multiple chemokine ligands.

Given the redundancy in ligands for CCR1 and CCR5, we hypothesize that it is essential for a therapeutic aimed at blocking migration induced by these receptors to bind and inhibit their relevant chemokine ligands. For instance, as CCL3, CCL4, and CCL5 have been found to be present at the sites of inflammation in RA and MS, inhibiting only a single chemokine will still allow for alternate chemokine ligands to act on the receptors. Conversely, as both CCR1 and CCR5 have been indicated in disease pathogenesis, blocking only a single receptor may not provide sufficient inhibition of chemotaxis as the other receptor may still respond to chemokines present at the site of inflammation. Presumably to overcome this redundancy, several pox viruses have evolved pan-chemokine binders that are capable of binding and inhibiting the activity of a large number of chemokines.

It is of particular interest that MAb d5d7 and vCCI were found to be equipotent inhibitors of chemotaxis of CCR1 and CCR5-transfectants upon stimulation with PBMC-conditioned media which contain a broader array of CCR1 and CCR5 chemokine ligands beyond CCL3, CCL4, and CCL5. This suggests that, while it may not share vCCI’s binding breadth, MAb d5d7’s more restricted binding repertoire is sufficient to inhibit relevant CCR1 and CCR5 ligands in an inflammatory setting. While we initially set out to develop an antibody that mimicked a portion of the binding breadth of a viral pan-chemokine binder, vCCI, we did not explicitly select for antibodies that directly mimicked the vCCI-chemokine binding interaction. Because of this, we were surprised to find that our MAb d5d7 epitope appears to overlap with that of vCCI. However, despite the preliminary evidence that both vCCI and MAb d5d7 appear to bind to similar regions of CCL3, CCL4, and CCL5 with high affinity, MAb d5d7 does not display the broad binding promiscuity that is characteristic of vCCI, indicating differences in the binding interactions. To better understand this, experiments are currently underway to reveal the high-resolution structure of d5d7 with its chemokine ligands.

To our knowledge, MAb d5d7 is the only anti-chemokine antibody that recognizes three human chemokine ligands with high affinity. Previous efforts have yielded two antibodies displaying cross-reactivity against two human chemokines, anti-MCP1/2 and anti-CXCL9/10 antibodies targeting blockade of CCR2 and CXCR3 receptors, respectively. The anti-MCP1/2 antibody, 11K2, was obtained through immunizing mice with MCP1 and subsequent analysis indicated that it bound to both MCP1 and MCP2 with K_D_ value of 4.66 pM and 428 pM, respectively [66]. To identify a monoclonal antibody that displayed cross-reactivity against CXCL9 and CXCL10, Fagete and coworkers utilized a different method in which a scFV recognizing CXCL10 was identified from phage libraries [62]. Following affinity maturation experiments to improve binding to CXCL10 in combination with high-throughput screening to identify variants that also bound CXCL9, an scFv was identified that was capable of inhibiting both CXCL9 and CXCL10-induced migration of CXCR3 transfected cells with IC_50_ values of 40 nM and 713 nM, respectively. Our strategy for obtaining MAb d5d7 differed from those described above as we included all three desired target chemokines in both the immunization and affinity maturation strategies. This difference may account for the near equivalent binding affinities and *in vitro* inhibitory activities of MAb d5d7 against all three target chemokines compared with the more disparate affinities and potencies of the anti-MCP1/2 and anti-CXCL9/10 antibodies to their respective ligands.

In targeting multiple chemokines, we have developed a monoclonal antibody capable of binding CCL3, CCL4, and CCL5. Alternate strategies could also be considered towards the same end, such as bispecific antibodies, polyclonal antibodies as well as fusion proteins containing the chemokine binding loops of chemokine receptors fused to Fc domains. However, we chose to pursue the development of monoclonal antibodies given their proven track record as successful therapeutic molecules. As opposed to the other multi-chemokine binding monoclonal antibodies that have been described, MAb d5d7 displays unique and desirable characteristics. First, it is the only multi-chemokine binding MAb that binds chemokine ligands that interact with more than one chemokine receptor, providing it with a broader ability to block leukocyte trafficking. In addition, both immunization and affinity maturation strategies explicitly selected for binding to all three chemokines ultimately resulting in a therapeutic antibody that is highly potent against three chemokines *in vitro* and *in vivo*. We anticipate that this novel antibody will have broad therapeutic utility in the treatment of autoimmune diseases due to its ability to simultaneously neutralize multiple chemokines implicated in disease pathogenesis. As chemokines play a major role in many immune-mediated diseases, the successful neutralization of their activities may provide significant therapeutic benefit in the treatment of autoimmune and inflammatory disorders.
